# Interventions for Psychological Health of Stroke Caregivers: A Systematic Review

**DOI:** 10.3389/fpsyg.2019.02045

**Published:** 2019-09-06

**Authors:** Anna Panzeri, Silvia Rossi Ferrario, Giulio Vidotto

**Affiliations:** ^1^Psychology and Neuropsychology Unit, Istituti Clinici Scientifici Maugeri, Veruno, Italy; ^2^General Psychology Department, Università degli Studi di Padova, Padova, Italy

**Keywords:** caregiver, stroke, psychological health, rehabilitation, psychological intervention, CBT, health psychology

## Abstract

**Background:** The increasing number of stroke patients (SPs) requires informal caregivers to bear a high burden of responsibilities and heavy (di)stress. Moreover, these issues could lead to the development of serious psychological problems (e.g., depressive and/or anxious) that in turn could give rise to poor health-related quality of life outcomes. However, although the value of psychological interventions has been widely recognized for SPs, the scientific literature lacks an updated synthesis of interventions addressing the psychological health of their caregivers.

**Aim:** The aim of this review is to summarize the interventions for the psychological health of stroke caregivers and provide a resume of literature-based evidence of their efficacy.

**Method:** A literature review from 2005 to date was conducted in three online databases: PubMed, Scopus, and Google Scholar. Eligibility criteria for studies were (A) English language, (B) caregivers and patients aged 18 years or above, (C) SP's caregiver beneficiating of a specific intervention, and (D) outcome measures addressing depressive and/or anxiety symptomology, quality of life, well-being, or burden.

**Results:** Across the selected 45 studies, substantial differences are observable in three main categories: (a) type of intervention (b) techniques, and (c) operators. Interventions' advantages and results are discussed. Overall, studies using psychological techniques, such as cognitive-behavioral therapy, coping skill-training, and problem-solving therapy, showed their usefulness and efficacy in reducing the caregivers' depressive and anxious symptoms, and burden. Interventions led by psychologists and tailored to meet caregivers' specific needs showed more positive outcomes.

**Conclusion:** This review underlines the usefulness of psychological interventions aimed at reducing the psychological burden, such as anxious and depressive symptomatology, of SPs' informal caregivers. Hence, psychological interventions for caregivers should be integrated as part of the stroke rehabilitation process to improve informal caregivers' and patients' quality of life and well-being.

## Introduction

Stroke is the second-leading cause of death in adults and a major cause of disability in the world (Feigin et al., 2017). It often implies severe consequences for patients who continue to require assistance, which is mostly provided by informal caregivers, usually spouses or other family members (Pindus et al., 2018). Informal caregivers represent an invaluable resource for stroke patients, playing a key role both during and after the rehabilitation process (Visser-Meily et al., 2006). Caregivers are required to bear many responsibilities, sometimes changing their roles, which can be extremely difficult (Camak, 2015).

Caregiving burden is a broad and multidimensional concept including all the several adverse effects of caregiving on the psychological, physical, social, and financial functioning (Zarit et al., 1986; Byun and Evans, 2015). Given that caregivers experience significant personal changes and bear a multi-compounded “load,” the term “strain” is also used as these negative consequences often deeply modify the caregiver's feelings and behaviors (Lazarus, 1993; Vidotto et al., 2010; Rossi Ferrario et al., 2014). In fact, in physics, the term strain indicates the deformation of a structure caused by the simultaneous effect of both load and stress.

Literature highlighted that caregiver burden can be further distinguished in two main areas that caregivers usually face (Rigby et al., 2009). On one hand, the so-called “objective” area comprises practical, financial, and physical-health difficulties; on the other hand, caregivers cope with subsequent issues in the psychological and social area, such as depression, anxiety, poor well-being, and relational troubles (Camak, 2015; Rossi Ferrario et al., 2019). Overall these interconnected areas constitute the broader construct of caregiver burden (Gbiri et al., 2015). Among the “objective” area, providing the necessary care requires caregivers to balance the patient's needs and their own personal and professional life (McLennon et al., 2014). Caregivers may reduce their time at work or may be forced to completely leave their job, with evident consequences regarding social involvement and financial condition (Bauer and Sousa-Poza, 2015). Furthermore, patients' medical and physical treatments require expensive therapies and drugs that exacerbate economic difficulties (Rajan et al., 2016). Concerning the social area, the caregiver's role in the family may be modified as well as the relationship with the stroke survivor, particularly for the spouse (Revenson et al., 2016; López-Espuela et al., 2018). Depending on the patient's condition, also affectivity and sexuality may undergo consistent modifications (Anderson and Keating, 2017). The reduced physical, cognitive, and sexual functioning (e.g., decline in libido and sexual disorders), and the increased survivor dependency may force many couples to reevaluate and transform their relationship in light of the new post-stroke roles (Tamam et al., 2008; McCarthy and Bauer, 2015). Completing caregiving tasks entails a reduction in free time and social contacts, leading to progressive isolation (Ekwall et al., 2005; Ratti et al., 2017; Woodford et al., 2018). Concerning the psychological area, feelings of solitude, depression, and anxiety are very common among caregivers and are reflected in poor physical health (Perkins et al., 2013; Persson et al., 2015). Therefore, over-stressed caregivers may provide SPs low-quality care (Em et al., 2017) and increase costs on healthcare systems (Jennum et al., 2015).

Until 1990, caregiving literature mostly focused on impaired elders and on adults with severe mental illness, then it progressively focused on cardiovascular pathologies and cancer, the most common chronic illnesses of adulthood (Sales, 2003). On one hand, some family caregiving strains are common across several illnesses, high illness severity is associated with greater objective and subjective strains independently from the specific pathology (e.g., stroke, cancer, heart disease, Alzheimer, mental illness). Moreover, caregivers face greater difficulties when the patient's behavior changes for affective and cognitive impairments, otherwise, they cope better with patients' physical impairments that seem to be more easily manageable (Biegel et al., 1991). On the other hand, some issues are pathology specific: feelings of shame and stigma are burdens specific of caregivers of patients with mental illness (Muralidharan et al., 2016); cancer caregivers face high uncertainty and anxiety levels and, those of brain cancer in particular, face the most difficult emotional suffering (Sales et al., 1992; Kent et al., 2016). Caregivers of patients with intellectual disability and Alzheimer are required to provide more physical care (Chiao et al., 2015; Werner and Shulman, 2015), and children's caregivers show the highest worries about the patient's future (Brannan et al., 1997; Pinquart, 2018). Finally, stroke caregivers have to cope with variable levels of cognitive deficits and/or physical disability that imply considerable objective and subjective burden (Camak, 2015). Stroke caregivers are older than brain injury caregivers, thus they face specific challenges in rehabilitation and for their own health (Sinnakaruppan and Williams, 2002). Moreover, compared to caregivers of neurological patients, stroke caregivers are at a greater risk of developing worst physical and emotional health, indeed they reported higher levels of anxiety and depression (Chow et al., 2006). Despite these evidences, too little attention is still given to caregivers who may be hidden or silent patients themselves (Sambasivam et al., 2019). Moreover, caring for caregivers' psychological health could contribute to achieving better rehabilitation outcomes for patients (Teasell et al., 2000).

In the last few decades, we assisted to a growth in the number of interventions aimed at supporting stroke caregivers and improving their quality of life and well-being (e.g., Cheng et al., 2018; Goudarzian et al., 2018; Kootker et al., 2019). However, such interventions are often conducted and conceptualized from a medical-nursing perspective, involving more educational issues than psychological ones, which are too often neglected (Mores et al., 2018). Given that psychological intervention already reached promising results with caregivers of other medical conditions (Kwon et al., 2017), it could play a key role also in changing stroke caregivers' everyday life conditions and in improving caregivers' and patients' physical and psychological health (Silvestro et al., 2016; Ward et al., 2016; Wilz and Pfeiffer, 2017).

Given the complexity of caregivers' conditions, it is of primary importance to provide holistic support by addressing practical-physical needs as well as psychological and emotional ones.

However, previous evidence showed mixed effects of psycho-social interventions on the psychosocial aspects of caregivers (Visser-Meily et al., 2005; Brereton, 2007; Legg et al., 2011) and scientific literature lacks an updated synthesis of interventions addressing the psychological health of stroke caregivers.

The objective of this review was to help health professionals to answer clinical and practical questions in choosing and planning support interventions for improving the psychological health of caregivers of adult stroke survivors.

Thus, the specific research question of this review was to understand which type of interventions are the most suitable to improve the caregivers' psychological health, with which modalities, figures, and techniques.

This systematic review aimed at summarizing the literature published since 2005 concerning interventions to improve SPs' caregivers' psychological well-being. Furthermore, a critical point of view from a psychological perspective is provided.

## Methods

The guidelines recommended by the Joanna Briggs Institute (Aromataris and Munn, 2017) and Okoli and Schabram (2010) were followed.

### Search Strategy

A systematic literature search was conducted to identify the papers about non-pharmacological interventions to promote PSs' caregivers' psychological well-being. The most cited review about interventions for stroke caregivers was published in 2005 (Visser-Meily et al., 2005), thus this year was chosen as the starting point. Only peer-reviewed journal articles in English published since 2005 were retrieved from three online databases: Scopus, PubMed, and Google Scholar. Given the variety of terms employed to describe psychological interventions for PSs' caregivers, two sets of keywords were chosen to identify the pertinent papers. The first set assessed the target population (caregivers, family, and stroke patients' spouses). The second set specified the type of intervention or program (psychological, psychotherapy, etc.). A wildcard symbol (^*^) was employed to generalize keywords typically characterized by varying suffixes. The search was performed by inserting logical conjunctions (AND/OR) between the sets. Search areas included the “title,” “abstract,” and “keywords” fields. The first screening of articles was based on the title and the abstracts. In the case of uncertainty, the article's entire text was read.

### Inclusion Criteria

Eligibility criteria for studies concerned several aspects. The population included primary PSs' caregivers and their patients aged 18 years and above. The caregiver was enrolled in or at least taking advantage of an intervention addressing psychological or well-being outcomes. Such interventions were characterized by various approaches (psychoeducation, counseling, CBT, social support, or even training in nursing and caring skills) and took place in various formats and settings. Included study designs were randomized clinical trials, clinical trials, or uncontrolled trials with pre- and post-test measurement. When a comparison group was present, it should be an attention-control group, a waiting-list control group, or a control group with “usual care” or “no treatment.” Comparison groups or historical cohort groups were also included. The outcome measures for caregivers addressed various psychosocial outcomes, such as depressive or anxiety symptomology, emotional state, burden, strain, well-being, quality of life, satisfaction in caregiving, and stroke knowledge.

### Exclusion Criteria

According to this review aims, several articles were excluded given their non-relevance, such as studies concerning pathologies other than stroke, studies including medical-pharmacological treatments, and studies without detailed descriptions of caregivers' outcomes, such as feasibility and protocol studies.

### Data Extraction and Systematization

From each retrieved article, the following information was extracted: the study design, the target recipients, the type of intervention and its methodology, the presence of psychological techniques, the measurements employed, outcomes, and general findings. The screening, extraction and coding stage were performed by two authors, one author (AP) was strictly supervised by another one (GV). The methodological quality appraisal was independently conducted by two authors (AP and SR) by following the Joanna Briggs Institute guidelines (Aromataris and Munn, 2017) that already showed their suitability in this field (Cheng et al., 2014). In a later phase, results were categorized and presented in various macro-areas, and their weaknesses and strengths were then highlighted.

## Results

### Search Results

**Figure 1:**
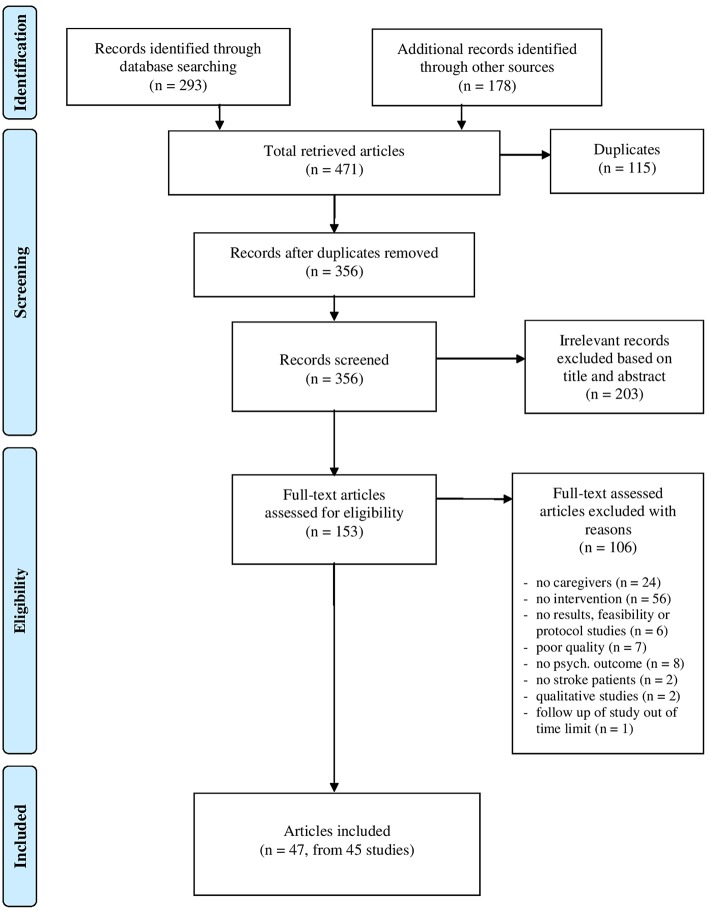
PRISMA Flow Diagram: study retrieval and selection.

The systematic search yielded 471 citations (Figure 1, PRISMA; Moher et al., 2015). After the removal of duplicates (*n* = 115), the remaining 356 articles were screened. Irrelevant records were excluded (*n* = 203); therefore, 153 articles were judged eligible and underwent a full-text assessment. Of those articles, 106 not fitting the review aims were excluded for various reasons: lack of intervention (*n* = 56); absence of caregivers (*n* = 24); absence of psychological outcomes (*n* = 8); poor quality (*n* = 7); protocol or feasibility studies (*n* = 6); absence of stroke patients (*n* = 2); qualitative studies (*n* = 2); follow up of a study conducted before 2005 (*n* = 1).

A total of 47 articles corresponding to 45 studies satisfied the inclusion criteria and were selected for the synthesis. Two of the 47 articles were a follow-up study (Shyu et al., 2010 follow up of Shyu et al., 2008) and an analysis of caregivers' data (Pierce et al., 2009) from the same original study that only analyzed patients' data (Steiner et al., 2008).

### Methodological Quality

Supplementary Table 1 summarizes results of the quality appraisal conducted according to the guidelines of the Joanna Briggs Institute (Cheng et al., 2014; Aromataris and Munn, 2017). Two authors independently evaluated the methodological quality and the risk of bias of the reviewed studies showing high agreement (Cohen's Kappa statistic = 0.90), disagreements were resolved discussing with the third author. Among the 45 studies, concerns on the risk of bias were mostly related to lack of: control group; randomization; blinding of allocators, participants, and assessors. However, the largest part of the included studies applied strong methodological designs, such as RCT, and large sample sizes: studies with these characteristics are considered more accurate and reliable. Overall, the included studies showed an acceptable to high quality level and low risk of bias.

#### Study Design

A control group was present in most of the studies (*n*_*tot*_ = 39), most were randomized control groups to lower the risk of selection bias (RCT studies; *n* = 30), followed by some not randomized (quasi RCT; *n* = 7) or were historical control groups (*n* = 2). Control groups included various forms: waiting list (*n* = 4), attention-control group minimizing performance bias (*n* = 5), and no treatment/treatment as usual (*n* = 30).

In the other studies the control group did not exist at all (*n*_*tot*_ = 6), such as quasi-experimental single group pre-post-test studies (*n* = 5) and a historical cohort study (*n* = 1).

### Study Characteristics

#### Country

This review included 45 studies conducted from 2005 to 2019 involving 5,038 informal caregivers. Study characteristics are described in Table 1. A total of 14 countries were represented: the USA (*n* = 10), the United Kingdom (*n* = 6), Germany (*n* = 4), China (*n* = 4), Australia (*n* = 5), Sweden (*n* = 3), South Korea (*n* = 4), Canada (*n* = 2), Iran (*n* = 1), the Netherlands (*n* = 2), Norway (*n* = 1), Turkey (*n* = 1), Portugal (*n* = 1), and Thailand (*n* = 1).

**Table 1:** Characteristics and results of the 45 studies ordered by relevance of positive psychological outcomes (descending).

**References**	**Study/control group (CG)/type control**	**Setting/target/*n*° caregiver**	**Operator(s)**	**Intervention type**	**Psych-Tech**.	**Delivery: via/setting/tailoring**	**Tools and results**
Wilz and Barskova, 2007	Historical cohort design/CG (*n* = 51), Not Random/Usual Care	Center/Car/*n* = 124	Psychologist	CBT group	Yes	Face/Group/Tailored	BDI^*^, BAI^*^, WHOQOL-BREF^*^
Ward et al., 2016	Quasi experimental–Pre-Post design/No CG	Center/SPandCar/*n* = 198	Psychologist	CBT group	Yes	Face/Group/Tailored	BDI^*^, HADS-D, HADS-A^*^, OCBS
Mei et al., 2018	RCT/CG (*n* = 28)/Usual care	Home/Car (*n* = 22), Dyad (*n* = 25)/*n*_tot_ = 83	Psychologist	Modified remiscence therapy	Yes	Face/Individual/Tailored	PAC^*^, SWLS^*^, CBI^*^
King et al., 2007	Historical cohort design/CG (*n* = 15), Not random/Usual care	From acute (Rehab.) to community/Car/*n* = 25	Nurse	CBT, problem solving	Yes	Face/Individual/Tailored	CES-D^*^, POMS-SF-T^*^, BCOS, PCS^*^
Graf et al., 2017	Historical cohort design/No CG	Home/Car/*n* = 70	Nurse	Problem-Solving, education, and support	Yes	Web + Tel/Individual/Tailored	CES-D^*^, ZBI^*^
Pfeiffer et al., 2014	RCT/CG (*n* = 62)/Usual care	Home/Car/*n* = 122	Psychologist	Problem solving	Yes	Face + Tel/Individual/Tailored	CES-D^*^, SPSI–R:S, LTS^*^, SCQ, GBB−24^*^
King et al., 2012	RCT/CG (*n* = 119)/Waiting list	From acute to community/Car/ *n* = 255	Psychologist, nurse	CBT: Problem-Solving, coping, psychoeducation, relaxation	Yes	Face + Tel/Individual/ Tailored	CES-D^*^, BCOS^*^, PH1-ad hoc^*^
Goudarzian et al., 2018	RCT/CG (*n* = 76)/Usual care	Home/Car/*n* = 152	Nurse	Telephonic counseling	Yes	Tel/Individual/Tailored	BDI, BAI^*^
Smith et al., 2012	RCT/CG (*n* = 19)/Attention control group	Home/Dyads/*n* = 38	Nurse	Psychoeducation, support		Web-Based/Individual/Tailored	CES-D^*^, RSES, MOS-SSS, PM
Fens et al., 2014	Quasi RCT/CG (*n* = 33), not random/Usual care	Home/Car/*n* = 74	Nurse	Psychoeducation		Face/Individual/Tailored	HADS-D^*^, HADS-A, LiSat-9, CSI
Perrin et al., 2010	RCT/CG (*n* = 34)/Usual care	Home/Car/*n* = 61	Nurse	Skill Development, education, and problem solving	Yes	Face + Web—Using videophone, Technology/Individual/One size	CES-D-10, CSI^*^
Cheng et al., 2018	RCT/CG (*n* = 64)/Usual care	From acute to community/Car/*n* = 142	Nurse	Psychoeducation, coping	Yes	Face + Tel/Individual/Tailored	CES-D, PSI^*^, CSI^*^, SSQ-6^*^; CCS, FAD-GF, SF-12
Bakas et al., 2009	RCT/CG (*n* = 19)/Attention-Control group	Home/Car/*n* = 40	Nurse	Psychoeducation		Tel/Individual/Tailored	PHQ-9, LOT-R^*^, BCOS, SF-36GH
Araujo et al., 2018	Quasi RCT/CG (*n* = 89) not random/Usual care	Home/Car/*n* = 174	Nurse	Nursing skill training		Face + Tel/Individual/Tailored	QASCI^*^, ECPICID-AVC^*^, SF-36GH, SF-36MH^*^
Inci and Temel, 2016	RCT/CG (*n* = 36)/Usual care	From Acute to Community/Car/*n* = 70	Nurse	Social support program, Psychoeducation		Face/Mixed/One Size	FIRA-G-FS^*^, FIRA-G^*^
Burton and Gibbon, 2005	RCT/CG (*n* = 89)/Usual care	Home (discharge destination)/SPandCar/*n* = 176	Nurse	Nursing support		Face + Tel/Individual/Tailored	CSI^*^
Chang et al., 2013	Quasi Exp. Pre-Post design/No CG	Rehab. Hosp. (Adult Day Care)/Dyad/*n* = 19	Nurse	Psychoeducation		Face/Mixed/Tailored	CSS^*^
Kim et al., 2012	Quasi RCT/CG (*n* = 31) not random/Usual care	From acute to community/Car/*n* = 73	Nurse	Psychoeducation, social support		Tel/Mixed/Tailored	FCB^*^
Oupra et al., 2010	Quasi RCT/CG (*n* = 70) not random/Usual care	Hospital/Car/*n* = 140	Nurse	Psychoeducation, support		Face + Tel/Mixed/One size	GHQ-28^*^, CSI^*^
Mores et al., 2018	Quasi Exp. Pre- Post test design/No CG	Center/Car/*n* = 42	Other therapists	Problem solving, coping	Yes	Face/Group/Tailored	BCOS^*^, OCBS^*^
Kootker et al., 2019	RCT/CG (*n* = 27)/Attention control group	Center/Pz—Car indirect/*n* = 42	Psychologist	CBT, Psychoeducation, relaxation	Yes	Face/Individual	HADS-D, HADS-A, IEQ-BI-W^*^, CSIGHQ^*^
Bunketorp-Käll et al., 2017	RCT/CG (*n* = 32)/Waiting list	Center/Pz—Car indirect/*n* = 106	Other therapists	Multimodal (Rhythm or Horse-Riding) Therapy		Face/Individual/One size	LISS^*^
Kim and Kang, 2013	Quasi RCT/CG (*n* = 14) not random/Usual care	Hospital/SPandCar/*n* = 28	Other therapists	Art mediated therapy		Face/Group/One size	PIL^*^
Bakas et al., 2015	RCT/CG (*n* = 131)/Attention control group	Home/Car/*n* = 254	Nurse	Psychoeducation, nursing skill training		Tel/Individual/Tailored	PHQ-9, SS-SSQOL proxy, BCOS
Robinson-Smith et al., 2016	RCT Pilot/CG (*n* = 5)/Usual care	Home/SPandCar Dyad/*n* = 10	Nurse	Psychoeducation, cognitive coping skills	Yes	Face/Individual (Dyad)/Tailored	CES-D, DCI, DCI-P^*^
Shyu et al., 2008 (Follow Up: Shyu et al., 2010)	Cluster RCT/CG (*n* = 86)/Usual Care	Hospital/Car/*n* = 158	Nurse	Psychoeducation, nursing: skill training and counseling		Face + Tel/Individual/Tailored	CNS^*^, PCS, SF-36 (SF-36)
Ostwald et al., 2014	RCT/CG, Attention control group	Home/Dyad/*n* = 159	Other therapists	Psychoeducation, nursing: counseling and skills		Face + Web (Mail)/Individual/Tailored	GDS, F-COPES, ZBI, PSS, MOS-SSS^*^, MS, PCS, SF-36^*^
Bishop et al., 2014	RCT/CG, Usual care	Home/Dyad/*n* = 49	Other therapists	Problem solving, psychoeducation	Yes	Tel/Individual/	GDS, FAD^*^, PCS1^*^, PCS2, FAI^*^
Torp et al., 2008	Quasi Exp. Pre-Post-test design/No CG	Home/Car/*n* = 19	Nurse	Psychoeducation, social support, nurse-counseling		Face + Web/Group/Tailored	RSS, FFCS^*^, GHQ-20
Cameron et al., 2015	RCT/CG (*n* = 10)/Usual care	From acute to community/Car/*n* = 31	Other therapists	Psychoeducation, Nurse-Counseling		Face + Tel/Individual/Tailored	CES-D, PANAS, MOS-SSS^*^, PM^*^
Kim et al., 2013	RCT/CG (*n* = 18)/Usual care	Home/Dyad/*n* = 30	Other therapists	Psychoeducation		Web/Individual/Tailored	CGMS^*^
Eames et al., 2013	RCT/CG (*n* = 30)/Usual care	From acute to community/SPandCar/*n* = 61	Occupational therapists	Psychoeducation		Face + Tel/Individual/Tailored	HADS-D, HADS-A, CSI, SE-ad hoc^*^, KSQ-*ad hoc*
Draper et al., 2007	RCT/CG (*n* = 11)/Waitlist control	Center/SPandCar/*n* = 39	Psychologist + other	Psychoeducation	Yes	Face/Group/One size	QLQ, RSS-20, MSRA, GHQ-28^*^
Marsden et al., 2010	RCT/CG (*n* = 8)/Waitlist control group	Center/SPandCar/*n* = 17	Multidisciplinary team	Psychoeducation		Face/Group/	HIS-E^*^, CSI
Louie et al., 2006	Quasi Exp. Pre-Post design/No CG	Rehab. Hosp./SPandCar/*n* = 32	Other therapists	Stroke education		Face/Group/One size	RSS, SKT^*^, SF-36
Franzén-Dahlin et al., 2008	RCT in blocks of 10/CG (*n* = 50)/Usual care	Hospital/Car/*n* = 100	Nurse	Psychoeducation		Face/Group/One size	SOC, KS-ad hoc^*^, CPRS-S-A
Johnston et al., 2007	RCT/CG (*n* = 85)/Usual care	Home/SPandCar/*n* = 160	Nurse	Psychoeducation, CBT	Yes	Face + Tel/Individual/One size	HADS-D, HADS-A, RLOC, SF-36
Tilling et al., 2005	RCT/CG (*n* = 170)/Usual care	Home/SPandCar/*n* = 340	Nurse	General support		Face + Tel/Mixed	HADS-D, HADS-A, PSS-C, CSI
Pierce et al., 2009 (original: Steiner et al., 2008)	RCT/CG (*n* = 37)/Usual care	Home/Car/*n* = 103	Nurse + other	Stroke Education, social support		Web/Group/One size	CES-D, SWLS (FS-*ad hoc*, PH-*ad hoc*)
Forster et al., 2009	RCT/CG (*n* = 49)/Usual care	Home/SPandCar/*n* = 106	Nurse	Psychoeducation		Face + Tel/Individual	CSI, GHQ-28
Björkdahl et al., 2007	RCT/CG = 15/ Usual care	Home/SPandCar/n = 35	Nurse + other	Nursing: counseling and skills, psychoeducation		Face/Individual/Tailored	CBS
Larson et al., 2005	RCT/CG = 50/ Usual care	Hospital/Car/n = 100	Nurse	Stroke education, support		Face/Group	QOL-VAS, LISS
Grasel et al., 2005	Quasi RCT/CG = 35/ Usual care	From acute to community/SPandCar/*n* = 71	Nurse	Nursing skill training, Psychoeducation		Face + Tel/Mixed/Tailored	D-S, BSFC, GSL-24
Hirsch et al., 2014	Quasi RCT/CG = 19/ Usual care	Rehab. Hosp/Car/*n* = 52	Nurse	Nursing skill training, Psychoeducation		Face/Individual	GDS, BSFC
Forster et al., 2013	RCT/CG = 478/ Usual care	Hospital/Car/n = 928	Multidisciplinary team	Nursing skill training, Psychoeducation		Face/Individual	CBS

* *Statistically significant positive outcome (p < 0.05); RCT, randomized controlled study; CG, control group; Car, Caregiver; SP, Stroke Patient; Dyad, caregiver and patient considered together; ACS, Appraisal of Caregiving Threat Subscale ACS); BCOS, Bakas Caregiving Outcomes Scale; BDI, Beck Depression Inventory; BSFC, Burden Scale for Family Caregivers; CBS, Caregiver Burden Scale; CCS, Caregiving Competency Scale; CES-D, Center for Epidemiologic Studies-Depression; CGMS-6, Care Giving Mastery Scale; CNC, Competing Needs Checklist; CPRS-S-A, Comprehensive Psychopathological Rating Scale–Self-Affective; CRQ, The Caring for Relatives Questionnaire; CSI, Caregiving Strain Index; CSS-15, Caregiving Satisfaction Scale; DCI, Dyadic Coping Instrument; DCI-P, Positive Dyadic Coping; D-S, Zerssen Depression Scale; ECPICID-AVCI, Skills Scale of Informal Caregivers of Dependent Older People Post-stroke; FAD, Family Assessment Device; FAD-GF, Family Assessment Device, General Functioning subscale; FAI, Frenchay Activity Index; FCB, Family caregiver burden; F-COPES, Coping; FFCS, Family and Friendship Contacts Scale; FIRA-G, Family Index of Regenerativity and Adaptation-General; FIRA-G-FS, Family Strain; FS-ad hoc, Family Support; GBB-24, Giessen Subjective (physical) Complaints List; GDS, Geriatric Depression Scale; GHQ-28, General Health Questionnaire; GSL-24, Giessen Symptom List; HADS, Hospital Anxiety and Depression Scale; HIS-E, Health Impact Scale, emotion subscale; HMS, Health Motivation Scale; IEQ-BI-W, Involvement Evaluation Questionnaire, Worry Subscale; ISSI, Interview Schedule for Social Interaction (Perceived social support); KS-ad hoc, Knowledge of Stroke; KSQ, Knowledge of Stroke Questionnaire; LiSAt-9: Life Satisfaction Questionnaire; LISS, Life Situation among Spouses after the Stroke Event; LOT-R, Life Orientation Test Revised (optimism); LTS, Leisure Time Satisfaction questionnaire; MCSS, Modified Caregiver Satisfaction Scale; MOS-SSS, Medical Outcome Study Support Survey; MS, Mutuality Scale; MSRA-25, Measure of Social and Recreational Activities; OCBS, Oberst Caregiving Burden Scale; PANAS, Positive and Negative Affect Schedule; PCS, Preparation for Caregiving Scale; PCS1, Perceived Criticism Scale–perceived of subject (how critical they consider their family); PCS2, Perceived Criticism Scale–perceived of subject (how critical participants consider themselves to be of their family); PH-ad hoc, physical health; PH1-ad hoc, single item physical health; PHQ-9, Depression; PIL, Purpose in Life; PM, Pearlin Mastery Scale; POMS-SF-T, Profile of Mood States, tension anxiety; PSI, Problem Solving Inventory; PSS, Perceived Stress Scale; PSS-C, Pound (life and care) Satisfaction Scale for Carers; QASCI, Informal Caregiver Burden Assessment Questionnaire; QLQ, Quality of Life Questionnaire; QOL-VAS, quality of life visual analog scale; RLOC, Recovery Locus of Control Scale; RSES, Self-Esteem Scale; RSS, Relative Stress Scale; SBI−15R, Systems of Belief Inventory, Beliefs and Practices subscale; SCQ, Sense of Competence Questionnaire; SE-ad hoc, self-efficacy; SF-36, Health Short Form; SF-36GH, General Health Subscale; SKT, Stroke Knowledge Test; SOC, Sense of coherence short version; SPSI–R:S, Social Problem-Solving Inventory–Revised; SSQ-6, Social Support Questionnaire; SWLS, Satisfaction With Life Scale; SF-12, Health Survey; WHOQOL-BREF, Whorl Health Organization Quality of Life; W-BQ-12, Well-being Questionnaire; ZBI, Zarit Burden Inventory*.

#### Participants

Considering the 45 analyzed studies, 25 studies were specifically addressed to caregivers, 12 were targeted at caregivers and patients, 6 were addressed to caregivers and patients but as a dyad, and 2 studies included interventions for patients but reported indirect positive outcomes for caregivers (Bunketorp-Käll et al., 2017; Kootker et al., 2019).

The caregivers' mean age ranged from 44 (Oupra et al., 2010) to 73 years (Torp et al., 2008). The majority of them were female and spouses of stroke patients; only in a few studies the majority of caregivers were daughters (Shyu et al., 2008; Oupra et al., 2010).

### Intervention Description

Various types of interventions emerged, according to their outcomes, operators, techniques, contents, recipients, setting, timing, and delivery mode.

#### Objectives

Each study addressed a number of outcomes according to its conceptual and theoretical framework. The most frequent outcomes for caregivers concerned strain or burden (*n* = 27), depression (*n* = 22) and anxiety (*n* = 11), stress (*n* = 5), general health (*n* = 17), physical health (*n* = 3), somatic complaints (*n* = 3), social support (*n* = 12), quality of life and well-being (*n* = 11), and caregiving competency or mastery (*n* = 11). Some studies addressed outcomes concerning life changes, situation, or satisfaction (*n* = 9). Few studies outcomes concerned the family functioning (*n* = 3) or individual resources such as self-esteem, beliefs, and coping (*n* = 4). Also, some positive outcomes were addressed, such as satisfaction in caregiving (*n* = 3), leisure-time satisfaction, optimism, positive affect, and positive aspects of caregiving.

#### Operators

The complexity of stroke caregivers' needs often necessitates a multidisciplinary team. Therefore, operators who conduct interventions may have various professional qualifications. A non-trivial distinction can be drawn between studies including psychological operators, such as psychologists and/or psychotherapists (*n* = 7), and non-psychological operators (*n* = 38), such as nurses (*n* = 26), occupational therapists, and other professional therapists (*n* = 12; e.g., physiotherapists, speech therapists, family organizers).

#### Techniques

The largest part of the analyzed studies employed more than one technique, but a main distinction concerns the intervention's core, which included psychological and non-psychological techniques. Psychological techniques or therapies were specifically intended to improve the caregiver's psychological well-being by means of cognitive behavioral therapy (CBT; *n* = 8), coping-skill training (*n* = 4), and problem-solving-skill training (*n* = 6). The cognitive behavioral theory assumes the existence of a strong connection between events, cognitive beliefs, emotions, behaviors, and thus the individuals' psychological health (Beck, 1979; Dobson and Dozois, 2019; Giuntoli et al., 2019). Coping processes are the cognitive and behavioral efforts to cope with stressful situations and emotions (Lazarus and Folkman, 1984), coping strategies showed a deep connection with psychological health (Yu et al., 2013; Quinn et al., 2014). Problem-solving is a systematic approach toward problems, its process consists in breaking down problems in smaller pieces to easily manage and solve them, it showed successful in reducing symptoms of depression after stroke (D'Zurilla and Maydeu-Olivares, 1995; Mitchell et al., 2009).

Non-psychological techniques were aimed at indirectly decrease caregivers' burden by improving nursing skills and caregivers' competencies (*n* = 11). Indeed, caregivers often lack preparedness and those practical and basic nursing skills that are required (Kalra et al., 2004; Araújo et al., 2015; Araujo et al., 2018).

Other techniques that are not of strictly psychological pertinence, such as psychoeducation (*n* = 30), counseling (*n* = 1), and enhancement of social support and sharing (*n* = 14), were grouped together. Psychoeducation is an extremely useful technique that combines stroke education and psychological support (Smith et al., 2012; Fens et al., 2014; Cheng et al., 2018), its usefulness to relief distress has already been proved in several contexts (Alves et al., 2016).

#### Setting

Studies were conducted in a variety of settings, including a hospital in acute phase (*n* = 6), rehabilitation setting during the post-acute phase (*n* = 3), or in the patient's home (*n* = 21). Some studies accompanied caregivers during the *iter* from the acute unit to the discharge destination (*n* = 8). Other interventions were performed in non-specified designated centers (*n* = 7) suitable for group or individual treatments.

#### Delivery Mode

In the analyzed studies, the intervention took place in individual format (*n* = 28), group format (*n* = 11), or a mixed format (*n* = 6).

Intrinsically different modalities were chosen: face-to-face (*n* = 20), telephone (*n* = 5), Web (*n* = 4) or face-to-face, and distance interventions (*n* = 16). Among the Web interventions, all used at least a Website with stroke educational materials in written or video format (Kim et al., 2013). Graf et al. (2017) used websites with factsheets, self-management tools, a list of resources, and a glossary of medical terms. Other interventions used not only informational websites and educational videos but also online chat sessions among peers, and e-mailed professional support (Pierce et al., 2009; Smith et al., 2012).

#### Tailoring

Interventions could be specifically tailored according to participants' needs (*n* = 27) or delivered in a standard “one size fits all” form (*n* = 11). A baseline assessment of needs often drives tailored interventions (e.g., Pfeiffer et al., 2014).

### Outcomes

A detailed description of the effects of interventions is available in the last column of Table 1, which shows the significant and non-significant results of each study. Studies are ordered according to the relevance of their psychological outcomes.

At the top, there are studies that significantly improved the core of psychological issues, such as depression and anxiety symptoms, and the well-being area. Below there are studies with significant results on caregiver burden followed by those with improvements in family functioning and social aspects. Subsequently, there are studies with improvements in nursing skills and stroke knowledge (e.g., Louie et al., 2006; Franzén-Dahlin et al., 2008), followed by studies with non-significant results (Grasel et al., 2005; Larson et al., 2005; Tilling et al., 2005; Björkdahl et al., 2007; Johnston et al., 2007; Forster et al., 2009, 2013; Pierce et al., 2009; Hirsch et al., 2014).

According to the results described in Table 1, a detailed description of various types of outcomes is provided below.

#### Depression

The majority of studies with significant improvements in depression symptoms were led by psychologists using psychological techniques (Wilz and Barskova, 2007; King et al., 2012; Pfeiffer et al., 2014; Ward et al., 2016). In other studies, depression improvements were achieved by nurses often using psychological techniques, such as CBT and problem solving (King et al., 2007; Graf et al., 2017), or psychoeducation, counseling, and support (Smith et al., 2012; Fens et al., 2014).

Otherwise, in other studies conducted by nurses or other therapists, there were no significant improvements in depression despite the use of psychological techniques (Johnston et al., 2007; Perrin et al., 2010; Bishop et al., 2014; Robinson-Smith et al., 2016; Cheng et al., 2018; Goudarzian et al., 2018) or psychoeducation as well (Grasel et al., 2005; Bakas et al., 2009, 2015; Ostwald et al., 2009; Eames et al., 2013; Hirsch et al., 2014; Cameron et al., 2015). Further studies, conducted by non-psychological operators and not using psychological techniques, did not find any significant improvement in depression (Tilling et al., 2005; Pierce et al., 2009).

#### Anxiety

Studies with significant improvements in anxiety symptoms were led by psychologists using psychological techniques (Wilz and Barskova, 2007; Ward et al., 2016) or were led by nurses still using psychological techniques such as counseling (Goudarzian et al., 2018) and problem solving (King et al., 2007).

Non-significant anxiety improvements were obtained by non-psychological operators using general support (Tilling et al., 2005), psychoeducation (Eames et al., 2013; Fens et al., 2014), or even psychological techniques as behavioral treatment (Johnston et al., 2007).

#### Burden

Significant improvements in burden were reported by studies conducted by psychologists using psychological techniques (King et al., 2012; Mei et al., 2018; Kootker et al., 2019), by studies conducted by operators other than psychologists but still using psychological techniques (Perrin et al., 2010; Graf et al., 2017; Cheng et al., 2018; Mores et al., 2018), or nursing skill training (Araujo et al., 2018), nursing support (Burton and Gibbon, 2005), and psychoeducation (Oupra et al., 2010; Kim et al., 2012; Inci and Temel, 2016). Non-significant results on caregivers' burden and stress were found mostly in studies with non-psychological operators using non-psychological techniques (Grasel et al., 2005; Tilling et al., 2005; Louie et al., 2006; Björkdahl et al., 2007; Forster et al., 2013; Hirsch et al., 2014). Non-significant results were reported even when these operators used psychoeducation (Torp et al., 2008; Bakas et al., 2009, 2015; Forster et al., 2009; Ostwald et al., 2009; Marsden et al., 2010; Eames et al., 2013; Fens et al., 2014) or CBT (King et al., 2007). However, only one study with psychologists using psychological techniques reported non-significant burden improvements (Ward et al., 2016) and in another one where only psychoeducation was used (Draper et al., 2007).

#### Well-Being

In the well-being area (WA) were included various aspects, such as general well-being, life satisfaction, life situation, satisfaction with caregiving, positive affect, positive aspects of caregiving, and purpose in life.

Studies with significant outcomes in the WA were often conducted by psychologists using psychological techniques (Wilz and Barskova, 2007; Pfeiffer et al., 2014; Mei et al., 2018), by nurses using psychoeducation (Bakas et al., 2009; Chang et al., 2013), or by other therapists using mediational techniques (Kim and Kang, 2013; Bunketorp-Käll et al., 2017).

Non-significant results in WA were reported by nurse-led studies not using psychological techniques (Larson et al., 2005; Tilling et al., 2005; Franzén-Dahlin et al., 2008; Pierce et al., 2009; Fens et al., 2014; Cameron et al., 2015) and by only one study including a psychologist but only using psychoeducation (Draper et al., 2007).

#### Cognitive and Personal Skills

This category includes various skills, such as coping and problem-solving skills, caregiving mastery and preparedness, locus of control, self-efficacy, and self-esteem.

Significant results were found by studies using psychological techniques (King et al., 2007; Robinson-Smith et al., 2016; Kootker et al., 2019) or psychoeducation (Eames et al., 2013; Kim et al., 2013; Cameron et al., 2015).

A smaller number of non-significant results were reported by studies that used psychological techniques (Johnston et al., 2007; Pfeiffer et al., 2014; Cheng et al., 2018) rather than by studies that did not use them (Ostwald et al., 2009; Shyu et al., 2010; Smith et al., 2012).

#### Social Support

Significant improvements in social support were found by studies using psychological techniques (Cheng et al., 2018) or psychoeducation (Ostwald et al., 2009; Cameron et al., 2015), otherwise non-significant results were reported mostly by nurse-led studies using only psychoeducation (Draper et al., 2007; Smith et al., 2012; Bakas et al., 2015).

#### Family Functioning

Significant improvements in family functioning were reported by a nurse-led study using a social support program and psychoeducation (Inci and Temel, 2016), and by a study that used psychological techniques (Bishop et al., 2014). Non-significant results were reported by nurse-led study using psychological techniques (Cheng et al., 2018).

#### Physical Condition

Significant improvements in general health and somatic complaints were found by studies led by psychologists using psychological techniques (Draper et al., 2007; King et al., 2012; Pfeiffer et al., 2014) or by nurse-led studies using psychological techniques (Bishop et al., 2014), or psychoeducation and support (Ostwald et al., 2009; Oupra et al., 2010).

Otherwise, non-significant results in physical improvements were shown by nurse-led studies not using psychological techniques (Louie et al., 2006; Pierce et al., 2009; Araujo et al., 2018), or psychoeducation (Grasel et al., 2005; Torp et al., 2008; Bakas et al., 2009; Forster et al., 2009; Shyu et al., 2010). Nurse-led studies did not find significant improvements even when psychological techniques such as CBT (Johnston et al., 2007) or coping skill training (Cheng et al., 2018) were used.

#### Nursing Skills and Stroke Knowledge

Interventions with significant improvements in nursing skills and stroke knowledge were led by nurses and used nurse skill training (Araujo et al., 2018), stroke education (Louie et al., 2006), or psychoeducation (Franzén-Dahlin et al., 2008). However, non-significant results were reported by a similar intervention led by occupational therapists using psychoeducation (Eames et al., 2013).

## Discussion

After examining results, it is possible to draw some general considerations.

A variety of delivery conditions were found across studies taking place in various settings (e.g., hospital, home, center), in individual or group format. In general, the delivery may include different modalities: face-to-face interventions allow to establish a stronger alliance and seem to be preferable to those at a distance (by telephone or via the Web) due to the latter's lower personal involvement and commitment. However, tele-interventions already proved their efficacy across several fields, they represent a low-cost and promising method to give support to more caregivers as well as a suitable integration to extend the time efficacy of face-to-face interventions (Chi and Demiris, 2015; Aldehaim et al., 2016; Wentzel et al., 2016; Jackson et al., 2018).

Individual interventions are more focused and tailored to each subject's needs, but they are very expensive; on the other hand, group interventions are less expensive and provide participants social support inside the group (Schure et al., 2006) that offers a sense of belonging to a new community that alleviates feelings of loneliness (Ward et al., 2016).

As a matter of fact, we found tailored group interventions that considered the specific needs of the participants and individual interventions that were not tailored. Most of the effective studies were tailored to the participants (Lutz and Camicia, 2016). Among the studies without effective psychological results, only a few were tailored. Interventions specifically addressing the caregiver's needs were more focused and efficient.

However, the key mechanism of effective interventions seems then to rely on the intervention type's core rather than its delivery conditions.

Concerning the interventions' type, according to the results section it is possible to observe that interventions conducted from a nurse-medical perspective were usually led by nurses or other health professionals other than psychologists. These interventions often adopted nursing-skill training, stroke education, and provision of support. Most of these interventions did not significantly reduce caregivers' strain or improve their well-being (Grasel et al., 2005; Larson et al., 2005; Tilling et al., 2005; Björkdahl et al., 2007; Johnston et al., 2007; Bakas et al., 2009, 2015; Forster et al., 2009, 2013; Pierce et al., 2009; Eames et al., 2013; Bishop et al., 2014; Hirsch et al., 2014; Ostwald et al., 2014; Cameron et al., 2015; Robinson-Smith et al., 2016). However, some studies yielded significant results in improving stroke knowledge (Louie et al., 2006; Franzén-Dahlin et al., 2008) and eventually reducing general burden, without any significant result in main psychological outcomes, such as anxiety and depression (Burton and Gibbon, 2005; Oupra et al., 2010; Perrin et al., 2010; Kim et al., 2012; Araujo et al., 2018; Cheng et al., 2018; Mores et al., 2018). Only in one study nurse counseling lowered anxiety symptoms but not the depressive ones (Goudarzian et al., 2018). Some nurse-led interventions improved depression outcomes and most of them applied psychological techniques (King et al., 2007; Smith et al., 2012; Fens et al., 2014; Graf et al., 2017). Remarkable results emerged in nurse-conducted interventions adopting a family perspective (Bishop et al., 2014; Inci and Temel, 2016). They effectively improved the overall family functioning and distress, focusing on communication among the family members but not on individual feelings and personal experiences.

A further step in the treatment of psychological issues seems to be provided by psychological interventions. Indeed, as reported in Table 1, all the studies with psychological interventions and including a psychologist as the operator yielded significant results regarding caregivers' psychological outcomes, such as significant improvements in depression, anxiety, well-being area, and strain (Wilz and Barskova, 2007; King et al., 2012; Pfeiffer et al., 2014; Ward et al., 2016; Mei et al., 2018). This result also occurred for the intervention with augmented CBT mainly targeting patients but also showing positive effects for caregivers (Kootker et al., 2019). In particular, Wilz and Barskova (2007) proved the efficacy of a cognitive-behavioral intervention “*administered by clinical psychologists, unlike several other programs for family members which have been evaluated in previous studies”* in improving caregivers psychological health: “*During the intervention, the spouses should have learned several new strategies for coping with disease-related changes.”* Also, Pfeiffer and colleagues' findings (2014) “*illustrate the benefits caregivers may experience from frequent, therapeutic, and guided cognitive-behavioral interventions.”* Recently, Mei et al. (2018) stated that “*Modified Reminiscence Therapy (MRT) is a process in which caregivers recall their personally significant past experiences with stroke survivors… [MRT] can improve their sense of happiness, quality of life, and the ability to adapt to their current situation.”*

As above described, a broad spectrum of techniques was adopted among the reviewed studies.

Non-psychological techniques were effective in reducing general burden and increasing stroke knowledge, social support, and general health, but they did not specifically affect key psychological issues, such as depression. Not surprisingly, the most effective in improving psychological outcomes were the psychological techniques, such as CBT, problem-solving and coping-skill training, and psychotherapy (Gallagher-Thompson and Coon, 2007; Poritz et al., 2016). Indeed, cognitive-behavioral techniques, such as problem-solving and coping skill training, already showed their usefulness in health psychology also when applied to several pathologies (King et al., 2007; Losada et al., 2015; Pietrabissa et al., 2017), and also when delivered by non-psychological operators—nurses—in order to decrease caregivers' burdens (Perrin et al., 2010; Cheng et al., 2018; Mores et al., 2018). However, psychological techniques, such as counseling and behavioral techniques, may be ineffective when used by non-psychological operators (Johnston et al., 2007; Bishop et al., 2014). Indeed, depression improvements were reported in some nurse-led studies that employed CBT techniques (King et al., 2007; Graf et al., 2017), but such results were not consistent with findings of other similar studies (Bishop et al., 2014).

Concerning the health professional's qualification, literature showed that interventions for caregivers are largely conducted by non-psychologist figures, such as nurses, occupational therapists, and unspecified operators, and, without any doubt, may produce significant results in educational objectives and in lowering general caregivers' burden (King et al., 2012). However, across the reviewed studies, the effective interventions to reduce caregivers' psychological symptoms and distress were led by psychologists by means of specific psychological techniques (Rossheim and McAdams, 2010; Kneebone, 2016). This finding is simultaneously foreboding and promising because psychologists seem to represent a valuable resource whose usefulness is still underrated.

As a whole, the examined studies confirmed that managing stroke sequelae in caregivers requires multiple skills, both psychological and practical ones. Given the complexity of stroke caregivers' needs, multifaceted interventions should be planned to address their psychological health. As shown in results' section, interventions for caregivers are multifaceted and vary across heterogeneous types according to their outcome, content, technique, participants, operators, delivery conditions, and effectiveness.

Interventions conducted from a nurse-medical perspective represent valuable and effective programs to strengthen caregivers' skills, knowledge, and to lower their burden. Nevertheless, this kind of interventions did not produce fully satisfying results in improving the core of psychological outcomes, such as depression and anxiety.

However, across the reviewed studies, psychological techniques showed their usefulness and efficacy to improve various psychological outcomes in caregivers' psycho-physical health. In particular, psychological techniques' lead to better results when the operators are psychologists or psychotherapists. Indeed, when psychological techniques are used by non-psychological professionals, results are much more uncertain and heterogeneous.

At this purpose, psychologists are specifically trained professionals of mental health who may enhance the efficacy of interventions for stroke caregivers by using specific psychological techniques.

Thus, in order to improve stroke caregivers' psychological health, is desirable that psychologists and psychotherapists take part in caregivers' interventions by using specific psychological techniques, such as CBT, coping and problem-solving training, counseling, and also psychoeducation.

Caregivers should be prompted and educated to receive and seek psychological professional help given their (possible) unawareness and the difficulties they might have to face (Rossi Ferrario et al., 2017; Tang et al., 2018; Waters et al., 2018; Rossi and Mannarini, 2019). To significantly improve caregivers' psychological outcomes, more specific interventions are then required, conducted in a broader psychological framework by specific professionals, such as psychologists, psychotherapists, and psychiatrists, when needed (Atwood, 2010).

### Limits

This systematic review has some limitations. First, it specifically focused on stroke caregivers and, despite the existence of analogies with caregivers of other pathologies, its generalizability to other populations is limited. Second, the search strategy was performed across three extensive online databases but considering additional sources could have returned further references thus potentially expanding the reviewed studies. The higher the number of searched sources, the higher the validity of results. Moreover, using independent judges in the coding stages would have improved the validity of results. Finally, we preferred to provide a qualitative—and not quantitative—synthesis of the current literature given the heterogeneity across the retrieved studies. In fact, we included study with different designs (not only RCT), and with a variety of outcomes; these reasons limit the availability of circumstances for robust meta-analyses. Despite the limitations of qualitative systematic reviews are more than those of quantitative methods, the qualitative synthesis approach is gaining stronger importance in the scientific literature (Petticrew and Roberts, 2006; Okoli and Schabram, 2010). Results from studies with small sample size should be taken with caution. Considering the above limitations, results should be taken with a critical view.

### Further Research

As first, further research may provide a quantitative synthesis of the literature—which was beyond the aims of this review. Furthermore, future RCT studies may disentangle the effectiveness of different types of interventions for caregivers of SPs, also testing modern therapies and approaches. Hopefully, future research will continue studying efficient interventions to improve caregivers' psychological health.

### Uninvestigated Questions

Despite the evidence regarding the multifaceted caregiver role and its consequences noted in international literature for chronic diseases in general and stroke in particular, some important topics remain under-investigated in a transversal way. One of them is the specific needs of younger patients and caregivers, who live differently from older ones regarding employment, child care, life planning, and sexuality (Kuluski et al., 2014; Quinn et al., 2014; Richards et al., 2016). Many doubts remain, particularly regarding younger caregivers and their particular needs. Still too little is known, specific assessments, interventions, and RCT studies might clarify these issues (Dutrieux et al., 2016). The impact of patients' cognitive consequences and behavioral changes on the caregivers and the specific interventions to reduce them are yet to be studied.

Moreover, the social costs of stroke's consequences in term of caregivers' job loss and their physical and mental health impairment despite their role in saving community resources are unknown (Glavin and Peters, 2015). This knowledge gap may hamper the planning of interventions intended to help caregivers reduce their strain. Finally, the caregiving role's cultural significance requires deeper consideration before interventions are planned, further cross-cultural comparisons should be useful (Pharr et al., 2014). In this regard, the absence of studies in some countries may reflect little attention to caregivers' psychological conditions and a consequently underscored frailty (Pendergrass et al., 2017).

## Conclusions

Caregivers of SPs display several needs, and often suffer of poor psychological health, thus in literature rose the number of interventions aimed at improving their well-being.

Nurse-led interventions have shown positive results, but the cooperation of various health professionals may significantly enhance the overall well-being of caregivers. Indeed, the existing interventions could still be improved, in particular by using psychological and psychotherapy techniques. Furthermore, evidences showed that psychologists may significantly improve the psychological health of stroke caregivers, by reducing depression, anxiety, and burden. Indeed, according to results, better psychological outcomes were associated to interventions using psychological techniques, such as CBT, problem-solving, coping skill training, and counseling. Furthermore, results from retrieved studies highlighted the valuable role of psychologists in reducing psychological health issues and distress when compared to other professionals such as nurses. Psychologists are health professionals who may enhance the quality and effectiveness of interventions for stroke caregivers. In view of the results, future studies and interventions could include psychologists and psychological techniques to improve caregivers' psychological health.

From this review, emerges that caregivers are not alone in dealing with the several issues they might face, in fact, a growing number of researches is caring for their physical and mental health.

## Author Contributions

AP performed the systematic search and the methodological quality appraisal, structured results, and wrote a large part of the manuscript. SR contributed to quality appraisal and critically reviewed the manuscript with GV who supervised the whole process. All the authors approve the final version of the article.

### Conflict of Interest Statement

The authors declare that the research was conducted in the absence of any commercial or financial relationships that could be construed as a potential conflict of interest.
