# Supplementary material for: Interventions for Psychological Health of Stroke Caregivers: A Systematic Review
Source: Front Psychol. 2019 Sep 6;10:2045. doi: 10.3389/fpsyg.2019.02045 (PMC6743500; doi:10.3389/fpsyg.2019.02045)
Supplement: Supplementary file 1 [file Table_1.docx]

***Supplementary Material***

**Supplementary Table 1. Methodological quality appraisal according to the JBI guidelines**

| Study Reference | 1. Was there a control group? | 2. Was the assignment to treatment group truly random? | 3. Were participants blinded to treatment allocation? | 4. Was allocation to treatment groups concealed from the allocator? | 5. Were the outcomes of people who withdrew described and included in the analysis? | 6. Were those assessing outcome blind to the treatment allocation? | 7. Were the control and treatment groups comparable at entry? | 8. Were groups treated identically other than for the named interventions? | 9. Were outcomes measured in the same way for all groups? | 10. Were outcomes measured in a reliable way? | 11. Was appropriate statistical analysis used? |
| --- | --- | --- | --- | --- | --- | --- | --- | --- | --- | --- | --- |
| Wilz and Barskova 2007 | √ | × | na | na | √ | na | √ | √ | √ | √ | √ |
| Ward et al. 2016 | × | na | na | na | × | na | na | na | na | √ | √ |
| Mei et al. 2018 | √ | √ | × | √ | ? | √ | √ | √ | √ | √ | √ |
| King et al. 2007 | √ | × | na | na | √ | na | na | na | na | √ | √ |
| Graf et al. 2017 | × | na | na | na | √ | na | na | na | √ | √ | √ |
| Pfeiffer et al. 2014 | √ | √ | ? | √ | √ | √ | √ | √ | √ | √ | √ |
| King et al. 2012 | √ | √ | × | ? | ? | √ | √ | √ | √ | √ | √ |
| Goudarzian et al. 2018 | √ | √ | ? | ? | √ | ? | √ | √ | √ | √ | ? |
| Smith et al. 2012 | √ | √ | √ | √ | ? | √ | √ | √ | √ | √ | √ |
| Fens et al. 2014 | √ | × | × | × | × | ? | ? | √ | √ | √ | √ |
| Perrin et al. 2010 | √ | √ | × | √ | × | ? | ? | ? | √ | ? | ? |
| Cheng, Chair, and Chau 2018 | √ | √ | × | √ | ? | √ | √ | √ | √ | √ | √ |
| Bakas et al. 2009 | √ | √ | ? | ? | × | √ | √ | ? | √ | √ | √ |
| Araujo et al. 2018 | √ | × | ? | × | √ | ? | √ | √ | √ | √ | √ |
| İnci and Temel 2016 | √ | √ | √ | ? | √ | × | √ | √ | √ | √ | √ |
| Burton and Gibbon 2005 | √ | √ | × | √ | ? | √ | √ | √ | √ | √ | √ |
| Chang et al. 2013 | × | na | na | na | × | na | na | na | na | √ | √ |
| Kim et al. 2012 | √ | × | × | × | × | × | √ | × | √ | √ | √ |
| Oupra et al. 2010 | √ | × | × | × | √ | √ | √ | × | √ | √ | √ |
| Mores et al. 2018 | × | na | na | na | ? | na | na | na | na | √ | √ |
| Kootker et al. 2019 | √ | √ | ? | ? | ? | √ | √ | √ | √ | √ | √ |
| Bunketorp-Käll et al. 2017 | √ | √ | × | √ | na | ? | √ | √ | √ | √ | √ |
| Kyoung Kim and Don Kang 2013 | √ | × | ? | na | na | na | √ | √ | √ | √ | √ |
| Bakas et al. 2015 | √ | √ | √ | × | × | √ | √ | √ | √ | √ | √ |
| Robinson-Smith et al. 2016 | √ | √ | ? | × | × | ? | ? | √ | √ | √ | ? |
| Shyu et al. 2008 | √ | ? | ? | × | × | × | √ | √ | √ | √ | √ |
| Ostwald et al. 2014 | √ | √ | × | √ | × | √ | √ | √ | √ | √ | √ |
| Bishop et al. 2014 | √ | √ | × | ? | √ | √ | √ | √ | √ | √ | √ |
| Torp et al. 2008 | × | na | na | na | √ | na | na | na | na | √ | √ |
| Cameron et al. 2015 | √ | √ | × | √ | √ | √ | √ | √ | √ | √ | √ |
| Kim, Lee, and Kim 2013 | √ | √ | × | ? | √ | ? | √ | √ | √ | √ | √ |
| Eames et al. 2013 | √ | √ | × | √ | ? | √ | √ | √ | √ | √ | √ |
| Draper et al. 2007 | √ | ? | × | ? | ? | × | √ | √ | √ | ? | √ |
| Marsden et al. 2010 | √ | √ | × | √ | √ | × | √ | √ | × | √ | √ |
| Louie, Liu, and Man 2006 | × | na | na | na | ? | na | na | na | na | √ | √ |
| Franzén-Dahlin et al. 2008 | √ | √ | × | √ | √ | √ | √ | √ | √ | √ | √ |
| Johnston et al. 2007 | √ | √ | × | √ | √ | √ | √ | 1 | √ | √ | ? |
| Tilling et al. 2005 | √ | √ | × | √ | × | √ | √ | √ | √ | √ | √ |
| Pierce et al. 2009 | √ | ? | × | × | × | ? | √ | √ | ? | √ | √ |
| Forster et al. 2009 | √ | √ | × | √ | × | √ | √ | √ | √ | √ | √ |
| Björkdahl, Nilsson, and Sunnerhagen 2007 | √ | ? | × | × | na | √ | ? | √ | √ | √ | ? |
| Larson et al. 2005 | √ | √ | × | ? | × | × | √ | √ | √ | √ | √ |
| Grasel et al. 2005 | √ | × | × | × | √ | √ | √ | √ | √ | √ | √ |
| Hirsch et al. 2014 | √ | × | × | na | √ | × | × | √ | √ | √ | √ |
| Forster et al. 2013 | √ | √ | ? | √ | √ | ? | √ | √ | √ | √ | √ |

Note: JBI = Joanna Briggs Institute; √ = Yes; ? = Unclear; × = No; na = Not applicable.
